# Inhibition of pathogenic non-enveloped viruses by 25-hydroxycholesterol and 27-hydroxycholesterol

**DOI:** 10.1038/srep07487

**Published:** 2014-12-15

**Authors:** Andrea Civra, Valeria Cagno, Manuela Donalisio, Fiorella Biasi, Gabriella Leonarduzzi, Giuseppe Poli, David Lembo

**Affiliations:** 1 Department of Clinical and Biological Sciences, University of Torino, San Luigi Gonzaga Hospital 10043 Orbassano, Torino, Italy

## Abstract

Recent studies reported a broad but selective antiviral activity of 25-hydroxycholesterol (25HC) against enveloped viruses, being apparently inactive against non-enveloped viruses. Here we show that 25HC is endowed with a marked antiviral activity against three pathogenic non-enveloped viruses, i.e. human papillomavirus-16 (HPV-16), human rotavirus (HRoV), and human rhinovirus (HRhV), thus significantly expanding its broad antiviral spectrum, so far recognized to be limited to viruses with envelope. Moreover, here we disclose the remarkable antiviral activity of another oxysterol of physiological origin, i.e. 27-hydroxycholesterol (27HC), against HPV-16, HRoV and HRhV. We have also identified a much weaker antiviral activity of other oxysterols of pathophysiological relevance, i.e 7α-hydroxycholesterol, 7β-hydroxycholesterol, and 7-ketocholesterol. These findings suggest that appropriate modulation of endogenous production of oxysterols might be a primary host strategy to counteract a broad panel of viral infections. Moreover, 25HC and 27HC could be considered for new therapeutic strategies against HPV-16, HRoV and HRhV.

The oxysterols are a family of 27-carbon molecules originating from cholesterol oxidation by either enzymatic or non-enzymatic mechanisms. Compared to cholesterol, they contain an additional hydroxy, epoxide or ketone group in the sterol nucleus, and/or a hydroxyl group in the side chain^1^,^2^. Various oxysterols of enzymatic origin have long been studied, thanks to the physiological roles they play, for instance, in bile acid synthesis, steroid hormone biosynthesis, sterol transport, and gene regulation. In particular, oxysterols like 22-R-hydroxycholesterol (22OHC), 25-hydroxycholesterol (25HC), and 27-hydroxycholesterol (27HC) were shown to be very good ligands of Liver X Receptors (LXRs)^3^, nuclear receptors that function as master transcription factors in cell metabolism and proliferation, as well as in inflammation and immunity^4^,^5^. More recently, a still-growing body of evidence has supported the potential contribution of certain physiologically-relevant oxysterols to the pathogenesis and progression of major human chronic inflammation-associated diseases^6^ but also their involvement in innate and adaptive immunity^7^.

New and original emphasis on the beneficial effects of at least certain oxysterols has now been given by the demonstration that 25HC displays marked broad antiviral properties^8^,^9^: added at low micromolar concentrations (1–10 μM) to cultivated cells, this oxysterol was shown to inhibit the entry of vescicular stomatitis virus (VSV) and human immunodeficiency virus (HIV), as well as cell membrane fusion. It also inhibited the replication of several other DNA and RNA viruses, all having as common feature an outer lipid envelope covering their protein capsid^8^. 25HC is reported to exert antiviral activity only against enveloped viruses, and not against non-enveloped viruses, a conclusion that was drawn from its lack of antiviral effect when added to human embryonic kidney cells infected with adenovirus Ad5 or Ad19, a non-enveloped virus. Moreover, in the same report, antiviral properties were shown only to be exerted by 25HC, and not by the two other side chain cholesterol oxidation products tested, i.e. 22(R)hydroxycholesterol and 22(S)hydroxycholesterol^8^. The few studies on the antiviral effect of oxysterols that have appeared to date have focused on enveloped viruses, and essentially on the antiviral activity of 25HC, the gene coding for 25-hydroxycholesterol oxidase having been shown to be modulated by type I interferons^10^ and by lipopolysaccharides^11^. HCV replication in Huh7 hepatoma cells was significantly reduced when cells were transfected with the oxysterol-binding protein related-protein 4 (ORP-4), then incubated with 25HC^12^. Likewise, HBV infection of HepG2 cells transfected with the sodium taurocholate cotransporting polypeptide (NTCP) membrane transporter was significantly counteracted by cell preincubation with 25HC, a treatment that interfered with viral entry^13^. In the model of HBV infected HepG2 cells, 22-S-hydroxycholesterol and 7β-hydroxycholesterol also appeared to exert significant antiviral effect^13^.

In the light of this interesting emerging evidence of 25HC's antiviral properties, and of the intriguing fact that the effect apparently selectively targets enveloped viruses, it was deemed useful to address this hot topic via an additional and wider approach, i) by focusing for the first time on a panel of non-enveloped viruses, consisting of major human pathogens, namely human papillomavirus-16 (HPV-16), the etiologic agent of cervical carcinoma and other human malignacies^14^, human rotavirus (HRoV), the etiologic agent of severe gastroenteritis in infants^15^ and the human rhinovirus (HRhV), the major etiologic agent of the common cold, the most frequent infectious disease in humans^16^, and ii) testing the antiviral effect not only of 25HC but also of the whole panel of the most widely found oxysterols in human blood^17^, namely 7-ketocholesterol (7kC), 7α-hydroxycholesterol (7αHC), 7β-hydroxycholesterol (7βHC) and, above all, 27HC, another oxysterol of enzymatic origin like 25HC with high pathophysiological impact, especially on innate and adaptive immunity and inflammation^18^.

The results demonstrate that 25HC and 27HC exert a marked inhibitory activity against HPV-16, HRoV and HRhV.

## Results and Discussion

The picture emerging from reported studies^8^,^9^ was that 25HC might be a broad-spectrum but selective inhibitor of enveloped viruses, being apparently inactive against non-enveloped viruses. To explore whether this really was the case, this study investigated the antiviral activity of 25HC against HPV-16, HRoV, and HRhV, which were selected for their severe impact on human health, and as representatives of three families of non-enveloped viruses: *Papillomaviridae, Reoviridae*, and *Picornaviridae*, each containing significant human pathogens^14^,^15^,^16^. Moreover, the most widely found oxysterols in human blood, all but one not previously tested for their antiviral potential (i.e. 27HC, 7αHC, 7βHC, 7κC) were included in this study.

**Figure 1:**
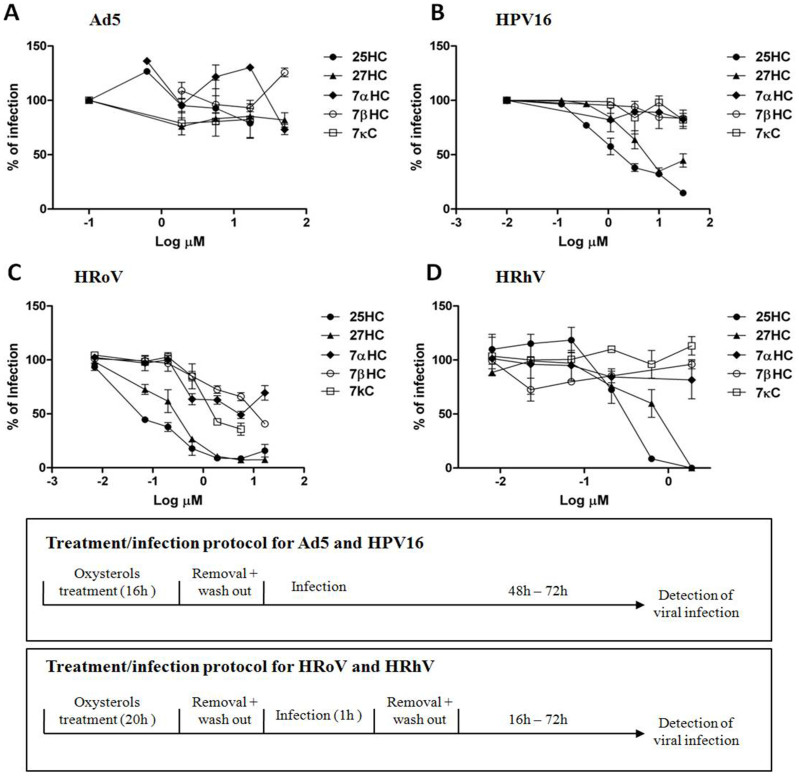
Antiviral activity of oxysterols against Ad5 (A), HPV16 (PsV) (B), HRoV (C) and HRhV (D). Cells were treated for 16 h (Ad5 or HPV-16 assays) or for 20 h (HRoV and HRhV assays) with increasing concentrations of oxysterols, and then infected. Viral infections were detected as described in the Methods section. The percentage infection was calculated by comparing treated and untreated wells. The results are means and SEM for triplicates.

A first set of experiments was performed by pretreating the cells with oxysterols for several hours before infection, as reported by Su-Yang Liu, (2013)^8^, in order to obtain data comparable to those reported in the literature. The results, shown in Figure 1A, demonstrate that neither 25HC nor the other tested oxysterols affected the infectivity of Ad5. These results confirmed those of the other study^9^. But, expanding this investigation to include other non-enveloped viruses, it was found that not only 25HC but also 27HC exerted a marked antiviral activity against HPV-16, HRoV and HRhV (Fig. 1, panel B, C, and D respectively) with EC_50_ values in the high nanomolar range for HRoV and HRhV, and in the low micromolar range for HPV-16 (Table 1, EC_50_ pretreatment column). Unlike these two side-chain cholesterol oxidation products, 7αHC, 7βHC and 7κC were found to be inactive against HPV-16 and HRhV, and barely active against HRoV. To exclude the possibility that the antiviral activity might depend on oxysterols' cytotoxic effects, a cell viability assay was performed on uninfected cells, challenged with the tested molecules under the same conditions as the antiviral assays. As shown in Table 1, neither 25HC nor 27HC exhibited any toxic effects in the range of concentrations employed (up to 150 μM) as is shown by the non-determinable 50% cytotoxic concentrations (CC_50_) and the favorable selectivity indexes (SI). Interestingly, when oxysterols were added to the cells at the same time of the virus inoculum (time zero) a full absence of any antiviral activity at all the tested concentrations appeared evident (Table 1). This finding ruled out a direct interference with virus-receptor interaction exerted by the oxysterols themselves. Moreover, it suggested that the antiviral activity was not mediated through a direct inactivation of viral particles by 25HC or 27HC. To verify this hypothesis, we performed a viral inactivation assay as described in the Methods section.

As shown in Table 2, the virus titers of samples treated with oxysterols did not significantly differ from those determined in samples exposed to the solvent only (i.e. ethanol) indicating that these molecules did not exert any direct effect at all on viral particles. Taken together, the results reported in Table 1 and Table 2 show that 25HC and 27HC exert their antiviral activity by targeting intracellular events involved in essential steps of the HPV-16, HRoV and HRhV replicative cycles. A multidisciplinary research approach is currently in progress aiming to elucidate the cellular effectors and/or the viral targets of 25HC and 27HC.

**Figure 2:**
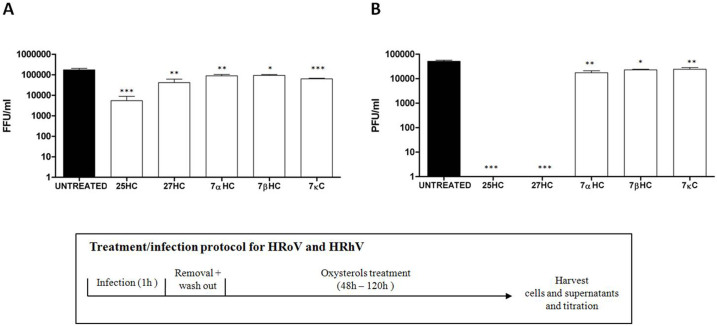
Virus yield reduction by oxysterols. Cells were infected with HRoV (A) or HRhV (B) and then treated with oxysterols (5 μM). When the cytopathic effect involved the whole monolayer of untreated wells (i.e. 48 hours post infection for HRoV and 120 hours post infection for HRhV), cells and supernatants were harvested together and titrated. The results are means and SEM for triplicates *** P < 0.001 **P < 0.01 * P < 0.05.

The antiviral activity of oxysterols was further investigated via a virus yield assay, a test condition that reflects more closely an ongoing productive infection *in vivo* being a stringent assay that allows multiple cycles of viral replication to occur before measuring the production of infectious viruses. Only those viruses that were susceptible to oxysterol inhibition in the previous experiments were tested (i.e. HRoV and HRhV); HPV-16 was not tested, since pseudovirus particles are unable to undergo a productive replicative cycle. In this set of experiments, cells were treated at a fixed oxysterol concentration (5 μM) after viral inoculum. The results, in Figure 2A, show that treatment with 25HC determined a significant (0.001 < p < 0.01) reduction in HRoV yield, while 27HC exerted modest, yet statistically significant, antiviral activity. Of note, both 25HC and 27HC completely abolished the replication of HRhV (Fig. 2 B). Moreover, under these experimental conditions, 7αHC, 7βHC and 7κC where slightly active against HRoV and HRhV. Taken together, these findings first of all extend the antiviral activity of 25HC to a panel of widespread non-enveloped viral pathogens, and demonstrate that the spectrum of antiviral activity of oxysterols could be broader than believed thus far. Moreover, this study discloses the antiviral potential of one of the most common oxysterols in the peripheral blood of healthy individuals, namely 27HC^13^, and, although to a lesser extent, of 7αHC, 7βHC and 7κC, also consistently detectable in human blood^13^.

Following the demonstration of antiviral properties of 25HC against a broad group of enveloped viruses^8^,^12^,^13^, the inhibition reported here, of non-enveloped viruses of primary global importance, by a panel of physiologically-relevant oxysterols, provides fresh and amplified support to the hypothesis that endogenous production of oxysterols might be a host strategy to counteract viral infections. In this connection, it is noteworthy that 27HC is generated by cholesterol 27-hydroxylase (CYP27A1), a mitochondrial cytochrome P450 oxidase present in various tissues and cells, particularly in the liver and macrophages^19^.

The EC_50_ values of 25HC and 27HC, calculated for HRoV, HRhV and HPV-16 (Table 1) are certainly higher than those found in the blood of healthy individuals, which are in the low nanomolar range^17^. However, they are relatively low compared to those detectable in inflammation-driven chronic disease processes, e.g. atherosclerosis^20^,^21^. This means that, theoretically, appropriate stimulation of their enzymatic production might be achievable, and in the light of the apparent absence of direct cytotoxic effects could be attempted (Table 1). How immune cells likely modulate the endogenous production of oxysterols remains entirely unknown, but will be a fascinating process to investigate. Overall, the therapeutic application of the antiviral properties of the panel of oxysterols studied here does not appear too distant a possibility.

## Methods

### Cell lines and viruses

African green monkey kidney epithelial (MA104) cells and human epithelial adenocarcinoma HeLa cells (ATCC® CCL-2™) were propagated in Dulbecco's modified Eagle's medium (DMEM) (Gibco-BRL, Gaithersburg, MD) supplemented with heat-inactivated 10% fetal bovine serum (FBS) (Gibco-BRL) and 1% antibiotic-antimycotic solution (Zell Shield, Minerva Biolabs GmbH, Berlin, Germany), at 37°C in an atmosphere of 5% of CO_2_. The 293TT cell line, derived from human embryonic kidney cells transformed with the simian virus 40 (SV40) large T antigen, was cultured as monolayer in medium in DMEM supplemented with heat-inactivated 10% FBS and Glutamax-I (Invitrogen, Carlsbad, CA) and nonessential amino acids. 293TT cells enable high levels of protein to be expressed by vectors containing the SV40 origin, due to overreplication of the expression plasmid.

Human rotavirus strain Wa (ATCC® VR-2018) was purchased from ATCC; the virus was activated with 5 μg/ml of porcine pancreatic trypsin type IX (Sigma, St. Louis, Mo.) for 30 minutes at 37°C, and propagated in MA104 cells using DMEM containing 0.5 μg of trypsin per ml as described elsewhere^22^.

Human rhinovirus 1A (ATCC® VR-1559) was purchased from ATCC. The virus was propagated in HeLa cells, at 33°C, in a humidified 5% CO_2_ incubator. When the full cytopathic effect (CPE) developed, cells and supernatants were harvested, pooled, frozen and thawed three times, clarified and aliquoted. Viruses were stored at −70°C. Rhinovirus titers were determined by the standard plaque method. Briefly, HeLa cells were seeded 2 days before infection in 96-well plates, reaching 60%–70% confluence at the time of infection. The viral suspension was serially diluted in DMEM supplemented with 2% fetal bovine serum and inoculated; the infected wells were incubated at 33°C for 1 hour, allowing viruses to attach and enter the cells. After this time, cells were washed with medium, and overlaid with a 1:1 combination of 1.6% SeaPlaque Agarose (BioWhittaker Molecular Applications) and 2 × DMEM medium (Gibco BRL) as described elsewhere^23^. The plates were incubated at 33°C for 3 days. After incubation, the plates were fixed and stained as described elsewhere^23^, the number of plaques formed was counted; viral titers were expressed in terms of plaque forming units per ml (PFU/ml).

Adenovirus 5 encoding GFP (GFP-Ad5) has a E1/E3 deletion that in HeLa cells results in a replication deficiency and it was purchased from Vector Biolabs (Philadelphia, PA, USA). The titer was 1.5 × 10^11^ PFU/ml in PBS with 5% Glycerol. The virus was stored at −80°C until use.

### HPV PsV production

Plasmids and 293TT cells used for pseudovirus (PsV) production were kindly provided by John Schiller (National Cancer Institute, Bethesda, MD). Detailed protocols and plasmid maps for this study can be seen at http://home.ccr.cancer.gov/lco/default.asp. HPV-16 PsVs were produced by methods described elsewhere. Briefly, 293TT cells were cotransfected with a bicistronic plasmid (p16 shell) expressing the papillomavirus major and minor capsid proteins (L1 and L2, respectively), together with a reporter plasmid expressing GFP. Capsids were allowed to mature overnight in cell lysate; the clarified supernatant was then loaded on top of a density gradient of 27 to 33 to 39% Optiprep (Sigma-Aldrich, St. Louis, MO) at room temperature for 3 h. The material was centrifuged at 28000 rpm for 16 h at 4°C in an SW41.1 rotor (Beckman Coulter, Inc., Fullerton, CA) and then collected by bottom puncture of the tubes. Fractions were inspected for purity in 10% sodium dodecyl sulfate (SDS)–Tris–glycine gels, titrated on 293TT cells to test for infectivity by GFP detection, and then pooled and frozen at −80°C until needed. The L1 protein content of PsV stocks was determined by comparison with bovine serum albumin standards in Coomassie-stained SDS-polyacrylamide gels.

### Reagents

25-, 27- 7α-, 7β-hydroxycholesterol (25HC, 27HC, 7αHC, 7βHC) or 7κ-cholesterol (7κC) (SIGMA) were dissolved in ethanol at concentrations ranging from 2.75 mM to 3 mM.

### Cell viability assay

Cells were seeded at a density of 5 × 10^3^/well in 96-well plates and treated the following next day with serially-diluted oxysterols (25HC, 27HC, 7αHC, 7βHC or 7κC) to generate dose-response curves. After 24 or 120 hours of incubation, cell viability was determined using the CellTiter 96 Proliferation Assay Kit (Promega, Madison, WI, USA), and following the manufacturer's instructions. Absorbances were measured using a Microplate Reader (Model 680, BIORAD) at 490 nm. The effect of oxysterols at different concentrations on cell viability was expressed as a percentage, by comparing absorbances of treated samples with those of the respective controls.

### HPV inhibition assay

HeLa cells were seeded 24 h in advance in 96-well tissue-culture-treated flat-bottom plates, at a density of 8,000 cells/well in 100 μl. The following day, cells were treated with serial dilutions of compounds. After 16 hours, compounds were removed and diluted PsV stocks (approximately 1 ng/ml L1) were added to the cells (this condition is indicated as “pretreatment” in Table 1). Alternatively the serial dilutions of oxysterols were added at the same time of the PsVs inoculum (this condition is indicated as “time zero” in Table 1). After 72 h at 37°C, the GFP-expressing infected cells were observed under an inverted Zeiss LSM510 fluorescence microscope (Zeiss, Oberkochen, Germany) and the percentages of infection were calculated by comparing GFP positive cells in treated and untreated wells.

### Rotavirus inhibition assays

The oxysterols' antiviral efficacy was determined by the focus reduction assay and the viral yield reduction assay. Assays of oxysterol inhibition of rotavirus infectivity were carried out with confluent MA104 cell monolayers plated in 96-well trays, as described elsewhere^24^. Cells were treated for 20 h at 37°C with 25HC, 27HC, 7αHC, 7βHC or 7κC, at concentrations ranging from 0.07 to 16.7 μM. Control samples were prepared by treating cells with culture medium supplemented with equal volumes of ethanol. Cells were later washed with medium, and rotavirus infection was performed at a multiplicity of infection (MOI) of 0.02 PFU/cell (this condition is indicated as “pretreatment” in Table 1). Alternatively the serial dilutions of oxysterols were added at the same time of HRoV inoculum (this condition is indicated as “time zero” in Table 1). After infection, cells were washed with medium and incubated at 37°C in a humidified incubator in 5% (vol/vol) CO_2_–95% (vol/vol) air. After 16 hours, infected cells were fixed with cold acetone-methanol (50:50), and viral titers were determined by indirect immunostaining.

To test the ability of oxysterols to inhibit multiple cycles of viral replication, MA104 were plated in 24-well trays, until they reached confluence. Cells were washed with medium and infected with trypsin-activated human rotavirus Wa (MOI 0.02). After one hour, cells were washed and fresh medium, supplemented with 0.5 μg/ml of porcine trypsin and containing different concentrations of oxysterols (ranging from 5.6 μM to 0.07 μM), was added. Infected cells and supernatants were harvested at 48 hours post-infection and titrated.

### Rhinovirus inhibition assays

The oxysterols' antiviral efficacy was determined by the plaque reduction assay and viral yield reduction assay.

HeLa cells were first seeded (at 8 × 10^4^ cells/well) in 24 well plates. The medium was removed from the plates before treatment with different concentrations of 25HC, 27HC, 7αHC, 7βHC or 7κC (ranging from 0.07 μM to 5.6 μM). After 20 hours of incubation (37°C, 5%CO_2_), medium with oxysterols was removed and infection was performed with 200 μl/well containing ca. 30 PFU of a stock of human rhinovirus 1A (this condition is indicated as “pretreatment” in Table 1). The infected cells were incubated at 33°C for 1 h, allowing the virus to attach and enter the cells. Alternatively the serial dilutions of oxysterols were added at the same time of HRhV inoculum (this condition is indicated as “time zero” in Table 1). After incubation, cells were washed with medium, and overlaid with a 1:1 combination of 1.6% SeaPlaque Agarose and 2 × DMEM. The plates were incubated at 33°C for 5 days. After incubation, the plates were fixed with 7.5% formaldehyde (Fluka) and stained with crystal violet (Sigma, St. Louis, Mo.). The number of plaques formed was counted.

To test the ability of oxysterols to inhibit multiple cycles of viral replication, HeLa cells were plated in 24-well trays, until they reached confluence. Each well was inoculated with 200 μl of a stock of human rhinovirus 1A containing ca. 30 PFU per well and incubated for 1 hour. After incubation, cells were washed with medium, and fresh medium was added (DMEM supplemented with 2% FBS) containing different concentrations of oxysterols (ranging from 5.6 μM to 0.07 μM). Infected cells and supernatants were harvested 5 days post-infection (when a complete CPE was visible), and titrated by using the plaque method as described above.

### Ad5 inhibition assay

HeLa cells were seeded 24 h in advance in 96-well tissue-culture-treated flat-bottom plates at a density of 8,000 cells/well in 100 μl. The following day, cells were treated with serial dilutions of 25HC, 27OHC, 7αHC, 7βHC or 7κC (ranging from 1.9 μM to 50 μM). After 16 hours, compounds were removed and Ad5 (MOI 0.02 PFU/cell) was added to cells (this condition is indicated as “pretreatment” in Table 1). Alternatively the serial dilutions of oxysterols were added at the same time of Ad5 inoculum (this condition is indicated as “time zero” in Table 1). After 48 h at 37°C, the GFP-expressing infected cells were observed under an inverted Zeiss LSM510 fluorescence microscope (Zeiss, Oberkochen, Germany) and the percentages of infection were calculated comparing GFP positive cells in treated and untreated wells.

### Virus inactivation assays

Oxysterols at respective EC_90_ dose or equal volume of ethanol were added to 10 ng of HPV, 2 × 10^5^ FFU/ml of HRoV or 2 × 10^5^ PFU/ml of HRhV and mixed in a total volume of 100 μl. The virus-compound mixtures were incubated for 0 hours or 2 hours at 37°C then serially diluted to the non-inhibitory concentration of test compound, and the residual viral infectivity was determined.

### Statistical analysis

All data were generated from duplicate wells in at least three independent experiments. Results of antiviral experiments are expressed as mean percentages ± standard error of the mean. Where possible, anti-viral effective concentration (EC_50_) values were calculated by regression analysis, using the dose-response curves generated from the experimental data, using GraphPad PRISM 5 (GraphPad Software, San Diego, California, U.S.A.). Viral yield reduction, in the presence and absence of oxysterols, was compared by analysis of variance (ANOVA) followed by a Bonferroni post-test, if *P* values showed significant differences, using GraphPad Prism 5 (GraphPad Software, San Diego, California, U.S.A.).

## Author Contributions

A.C. performed the antiviral assays against HRoV and HRhV and V.C. performed the antiviral assays against HPV and Ad5. A.C., V.C., G.P. and D.L. were involved in conception of this work. M.D., F.B. and G.L. gave scientific advices and contributed to a deep manuscript revision. D.L. supervised the work on the whole and provided laboratory means for the antiviral assays. All authors contributed substantially to the present work then read and approved the final manuscript.

## Figures and Tables

**Table 1:** Antiviral activities of oxysterols

Oxysterols	Virus	EC_50_* time zero (μM)	EC_50_* (95% C.I.)** 16/20 h pretreatment (μM)	CC_50_*** (μM)	SI****
**25HC**	Ad5	n.a.	n.a.	>150	n.a.
	HPV-16	n.a.	2.20 (1.61–2.99)	>150	>68.18
	HRoV	n.a.	0.05 (0.03–0.10)	>150	>3000
	HRhV	n.a.	0.29 (0.18–0.44)	>150	>517.2
**27HC**	Ad5	n.a.	n.a.	>150	n.a.
	HPV-16	n.a.	9.34 (5.07–17.19)	>150	>16.06
	HRoV	n.a.	0.25 (0.19–0.33)	>150	>600
	HRhV	n.a.	0.62 (0.43–0.89)	>150	>241.9
**7αHC**	Ad5	n.a.	n.a.	>150	n.a.
	HPV-16	n.a.	n.a	>150	n.a.
	HRoV	n.a.	n.a.	>150	n.a.
	HRhV	n.a.	n.a	>150	n.a.
**7βHC**	Ad5	n.a.	n.a.	35.93	n.a.
	HPV-16	n.a.	n.a.	35.93	n.a.
	HRoV	n.a.	n.a.	71.84	n.a.
	HRhV	n.a.	n.a.	35.93	n.a.
**7κC**	Ad5	n.a.	n.a.	82.22	n.a.
	HPV-16	n.a.	n.a.	82.22	n.a.
	HRoV	n.a.	n.a.	40.31	n.a.
	HRhV	n.a.	n.a.	82.22	n.a.

*EC_50_ half maximal effective concentration.

**C.I. confidence interval.

***CC_50_ half maximal cytotoxic concentration.

****SI selectivity index.

n.a. not assessable.

**Table 2:** Virus inactivation assay

Incubation time	Virus	Incubation condition	Viral titer* ± SEM **
**0 h**	HPV-16	Ethanol	3.2 × 10^6^ ± 0.4 × 10^6^
		25HC	3.4 × 10^6^ ± 0.4 × 10^6^
		27HC	3.3 × 10^6^ ± 0.1 × 10^6^
	HRoV	Ethanol	5.8 × 10^5^ ± 0.7 × 10^5^
		25HC	5.3 × 10^5^ ± 0.4 × 10^5^
		27HC	5.9 × 10^5^ ± 0.7 × 10^5^
	HRhV	Ethanol	1.5 × 10^5^ ± 0.2 × 10^5^
		25HC	2.1 × 10^5^ ± 0.1 × 10^5^
		27HC	2.3 × 10^5^ ± 0.3 × 10^5^
**2 h**	HPV-16	Ethanol	2.0 × 10^5^ ± 0.1 × 10^5^
		25HC	2.5 × 10^5^ ± 0.1 × 10^5^
		27HC	2.3 × 10^5^ ± 0.2 × 10^5^
	HRoV	Ethanol	4.5 × 10^5^ ± 0.2 × 10^5^
		25HC	4.2 × 10^5^ ± 0.1 × 10^5^
		27HC	4.9 × 10^5^ ± 0.2 × 10^5^
	HRhV	Ethanol	9.4 × 10^4^ ± 0.6 × 10^4^
		25HC	10.7 × 10^4^ ± 0.7 × 10^4^
		27HC	9.2 × 10^4^ ± 0.9 × 10^4^

*viral titer is expressed as FFU/ml for HPV-16 and HRoV and in PFU/ml for HRhV.

**SEM: standard error of the mean.
